# Non-human primate and rodent embryonic stem cells are differentially sensitive to embryotoxic compounds

**DOI:** 10.1016/j.toxrep.2014.11.016

**Published:** 2014-12-31

**Authors:** Lauren Walker, Laura Baumgartner, Kevin C. Keller, Julia Ast, Susanne Trettner, Nicole I. zur Nieden

**Affiliations:** a Department of Cell Biology & Neuroscience and Stem Cell Center, College of Natural and Agricultural Sciences, University of California Riverside, Riverside, CA 92521, USA; b Environmental Toxicology Graduate Program, College of Natural and Agricultural Sciences, University of California Riverside, Riverside, CA 92521, USA; c Fraunhofer Institute for Cell Therapy & Immunology, Perlickstrasse 1, 04103 Leipzig, Germany

**Keywords:** Osteogenesis, Embryonic stem cell test, Marmoset, Rhesus, Sensitivity, Embryotoxicity

## Abstract

Many industrial chemicals and their respective by-products need to be comprehensively evaluated for toxicity using reliable and efficient assays. In terms of teratogenicity evaluations, the murine-based embryonic stem cell test (EST) offers a promising solution to screen for multiple tissue endpoints. However, use of a mouse model in the EST can yield only a limited understanding of human development, anatomy, and physiology. Non-human primate or human *in vitro* models have been suggested to be a pharmacologically and pathophysiologically desirable alternative to murine *in vitro* models. Here, we comparatively evaluated the sensitivity of embryonic stem cells (ESCs) of a non-human primate to skeletal teratogens with mouse ESCs hypothesizing that inclusion of non-human primate cells in *in vitro* tests would increase the reliability of safety predictions for humans.

First, osteogenic capacity was compared between ESCs from the mouse and a New World monkey, the common marmoset. Then, cells were treated with compounds that have been previously reported to induce bone teratogenicity. Calcification and MTT assays evaluated effects on osteogenesis and cell viability, respectively. Our data indicated that marmoset ESCs responded differently than mouse ESCs in such embryotoxicity screens with no obvious dependency on chemical or compound classes and thus suggest that embryotoxicity screening results could be affected by species-driven response variation. In addition, ESCs derived from rhesus monkey, an Old World monkey, and phylogenetically closer to humans than the marmoset, were observed to respond differently to test compounds than marmoset ESCs. Together these results indicate that there are significant differences in the responses of non-human primate and mouse ESC to embryotoxic agents.

## 1 Introduction

In the United States, one in 28 babies carries congenital anomalies [1]. Although 50% of the causes for such birth defects are unknown, some may be traced back to involuntary environmental chemical exposure. There are more than 80,000 cataloged chemicals in the United States that may be released into the environment and most of them are inappropriately tested for safety. This lack of information is particularly concerning for sensitive populations such as pregnant women and children as adequate safety guidelines cannot always be confidently recommended. Furthermore, given that the developing fetus is especially sensitive to maternal environmental conditions and also that exposure during key points of development can lead to unique effects lasting through multiple generations [2], the potential embryotoxicity and teratogenicity of industrial compounds is of particular concern.

With appropriate data, acceptable exposure levels and actual safety of such products can be established for individuals that are most vulnerable to chemical exposure. Therefore, toxicology programs have been designed to identify toxicities that may potentially be encountered in human embryos. Under the worldwide trend for revision of chemical legislation, it will be necessary to test a large number of chemicals in a short time, which can only be achieved with predictive *in vitro* assays.

A step in the direction of animal sacrifice free embryotoxicity screen was taken when the classic embryonic stem cell test (EST) was first described [3], [4]. This assay relies on embryonic stem cells (ESCs) from the mouse and compares two important aspects of prenatal toxicity. First, the EST has revealed the differences in sensitivity of mouse embryonic stem cells (ESCs) to chemical entities compared to adult fibroblasts. Second, the test determines the ability of a chemical to inhibit the differentiation of the ESCs into a differentiated cell type of interest [5], [6].

Among the many birth defects, the ones that affect musculoskeletal tissues account for 5% of all infant deaths. Thus, skeletal toxicity has become a high priority screening phenotype and is currently integrated into the animal screens that assess general prenatal developmental toxicity (TG414, OECD) [7], [8], [9]. Assessing the inhibition of osteogenic differentiation of the ESCs, the EST may also be exploited to serve as predictor for developmental osteotoxicity [6], [10], [11], [12], [13], [14].

Despite the routine use of rodent models in research, the mouse model as used in the EST can only yield a limited understanding of human development, anatomy and physiology. Accordingly, human *in vitro* models are desirable from a pharmacological and pathophysiological standpoint. Indeed, ESCs from humans were established around the turn of the century [15]. However, due to ethical considerations, the legality of their use varies widely between countries. A solution comes with human induced pluripotent stem cells (hiPSCs), which are artificially created from somatic cells, and are therefore not ethically challenged, but it is yet unclear how their quality or differentiation potential measures up to bona fide hESCs. Therefore, to provide a legal and ethical alternative to countries, which have banned hESC research, we test here whether the sensitivity of non-human primate ESCs to a small set of classical and skeletal embryotoxic agents is similar to that of mouse ESCs in order to evaluate whether the inclusion of non-human primate cells into the EST would increase the reliability of safety predictions for human use.

## 2 Materials and methods

### 2.1 Murine ESC maintenance

Murine D3 embryonic stem cells (American Type Culture Collection, Rockville, MD, USA) were expanded in high glucose DMEM containing l-glutamine (Corning). Media additionally contained 15% batch-tested fetal bovine serum (FBS), 1% non-essential amino acids (NEAA), 50 U/ml penicillin, 50 μg/ml streptomycin, 0.1 mM 2-mercaptoethanol (all Invitrogen) and 1000 U LIF/ml (Millipore). Cells were routinely passaged every 2–3 days with 0.25% Trypsin-EDTA (Life Technologies).

### 2.2 Maintenance culture of non-human primate ESCs

Embryonic stem cells from the common marmoset (cjes001) were cultured in feeder-free conditions as described [16]. Rhesus ESCs (R366.4, WiCell Research Institute) were cultured on mouse embryonic fibroblast feeder layers as previously described [17], [18].

### 2.3 Osteogenic differentiation of ESCs

Murine ESCs were induced to differentiate *via* aggregation into embryoid bodies *via* hanging drops at 750 cells/drop, in the presence of control differentiation medium (CDM, mouse ESC maintenance medium without LIF [19]. Differentiating cells were replated on day 5 as a single cell suspension at a concentration of 50,000 cells/cm^2^ [20]. Differentiation of marmoset and rhesus ESCs was initiated from intact ESC colonies in non-adherent conditions as described [16], [17]. In brief, undifferentiated colonies were trypsinized with TrypLE (Invitrogen) into clusters of 20–30 cells. Approximately 100 such clusters were seeded in CDM to one bacteriological grade dish (100 mm diameter). Following 5 days of incubation, cell clusters were transferred onto cell culture plates coated with 0.1% gelatin at an approximate density of 10 cell clusters/cm^2^. On day 5 of differentiation, cells from all species received osteogenic differentiation medium containing the induction factors β-glycerophosphate (10 mM), ascorbic acid (25 μg/ml), and 1α,25-(OH)_2_ vitamin D_3_ (5 × 10^−8^ M) in CDM.

### 2.4 Test compounds

5-fluorouracil, *all-trans* retinoic acid, penicillin G (all Sigma) were selected as control test compounds as the teratogenic potential of each has been well established by previous *in vivo* and *in vitro* investigations [21]. Stock solutions were made in DMSO and diluted to test concentrations in respective cell culture media. Lithium chloride was obtained from Fluka and aluminum chloride was obtained from Sigma. Sodium chloride (Fisher Scientific), lithium acetate (Aldrich), sodium acetate (Sigma), and aluminum hydroxide (Sigma) were included as controls for lithium and aluminum activity. Untreated control cultures containing appropriate vehicle were also included. Osteogenic differentiation was considered valid if the control solvent yielded osteoblast differentiation levels comparable to that of untreated vitamin D_3_ induced osteogenic cultures.

### 2.5 Cytotoxicity assay

Cellular viability was evaluated with an MTT assay following 14 days of osteogenic induction as described [22]. In brief, 0.5 mg/ml MTT solution was added to the cultures and cells were incubated at 37 °C for 2 h. Reagent was then aspirated and cells were gently rocked in pre-warmed MTT desorb solution (0.7% SDS in 2-propanol) for 15 min. Absorbance of dissolved blue formazan product was measured spectrophotometrically at 570 nm with a 630 nm reference wavelength. Mitochondrial activity was normalized to solvent only controls and resulting percentages were graphed along the tested concentration range to construct a concentration–response curve. The half-maximal inhibitory effect (IC_50_) for each compound was subsequently established *via* linear interpolation of the curve.

### 2.6 Determination of Ca^2+^ content

#### 2.6.1 Alizarin Red S staining

Attached cells were washed with 1X PBS and fixed with 4% paraformaldehyde in 1× PBS and incubated at 4 °C for 1 h. Residual fixative was quenched *via* incubation with 100 mM glycine for 15 min at room temperature. Samples were then washed three times in 1× PBS and once in dH_2_O. Fixed cells were then subjected to a 0.5% Alizarin Red S staining solution for 5 min. Following three washes with dH_2_O, subsequent washes were performed with ascending ethanol concentrations (*i.e.* 70%, 80%, 90%, and 100%). Cultures were kept in 100% ethanol for acquisition of images.

#### 2.6.2 Quantification of calcium deposition

Cells were washed twice in 1× phosphate buffered saline (PBS) and lysed in a modified RIPA buffer (1% NP-40, 0.5% sodium deoxycholate, 0.1% sodium dodecyl sulfate in 1× PBS, pH 7.4). Each plate was incubated for 1 h at 4 °C with shaking to ensure complete cell lysis. Ca^2+^ concentration was measured against a set of standards using an Arsenazo III based spectrophotometric assay (Genzyme Diagnostics) at 650 nm as described [23]. The protein concentration in each sample was then measured against a set of standards using a Lowry spectrophotometric assay (Bio-Rad Laboratories) at 750 nm. Ca^2+^ content in each sample was then normalized to the respective protein concentration measured with the Bio-Rad DC protein assay reagent as described [23]. Calcium content was normalized to solvent only controls and concentration-response curves charted. The half-maximal inhibitory dose (ID_50_) for each compound was taken from linear interpolation of the curve.

### 2.7 Statistical analysis

Data significance was decided using a web-based one-way ANOVA and Tukey HSD *post hoc* test (http://faculty.vassar.edu/lowry/anova1u.html) or unpaired Student's *t*-test as appropriate. All results are represented as average of five independent replicates ± standard deviation.

## 3 Results

### 3.1 Osteogenic differentiation potential of marmoset ESCs

**Fig. 1:**
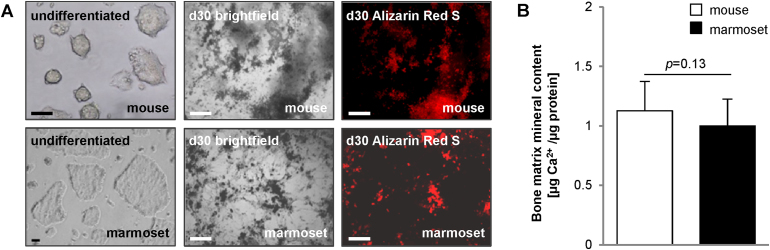
Osteogenic differentiation ability in mouse and marmoset ESCs. (A) Brightfield images and Alizarin Red S staining identifying mineralized calcium. (B) Quantification of calcium deposit in osteogenic cultures determined with Arsenazo III, *n* = 3, five technical replicates each ± SD. *p*-value was established with a Student's *t*-test.

ESCs from the marmoset, *Callithrix jacchus*, a New World monkey, have been previously derived [18], [24], [25] and been shown to be capable of producing osteoblasts that calcify their extracellular matrix [16]. Calcified extracellular matrices had been previously described as dark colored light-dense areas [26]. Those dark areas were observed in cultures from both mouse and marmoset ESCs *via* bright field microscopy on day 30 of osteogenic differentiation (Fig. 1A). Alizarin Red S staining of cultures confirmed the presence of calcified extracellular matrix in such dark areas. The overall amount of calcification between the marmoset and the mouse cells was comparable (Fig. 1B).

### 3.2 Differential sensitivity of mouse and marmoset ESCs to lithium derivatives and controls

Previous literature has suggested that lithium chloride, actively used in psychiatric pharmaceuticals, possesses the capacity for inducing skeletal teratogenicity [27]. In addition, our own research has suggested that lithium derivatives cause skeletal teratogenicity in certain concentration ranges [22]. To test the predictive aptitude of a non-human primate based EST for lithium derivatives, mouse and marmoset osteogenic ESC cultures were treated with lithium and aluminum compounds. Sodium chloride and lithium acetate served as a control for chloride in lithium chloride to ensure that observed effects were due to lithium activity. Sodium acetate was included as a control for acetate in lithium acetate.

**Fig. 2:**
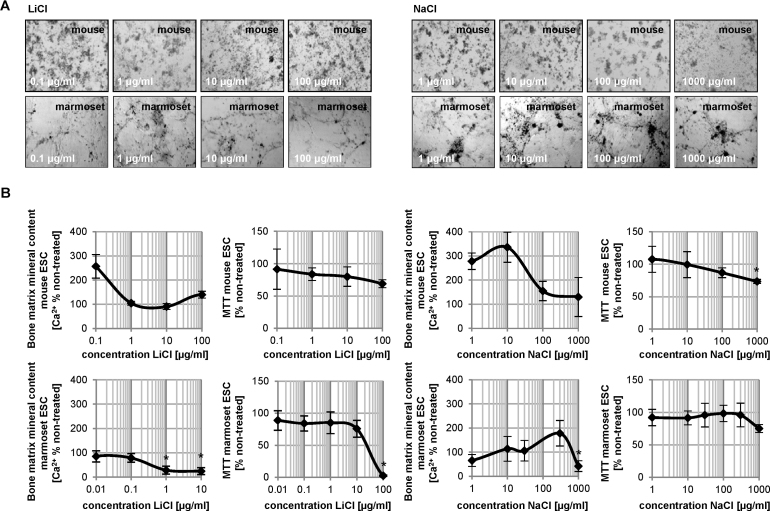
Cytotoxicity and bone mineral matrix assessment in mouse and marmoset osteogenic cultures treated with lithium and sodium chloride. (A) Morphology of cultures. (B) Reduction in survival rate and calcium content is given as percentage of non-compound treated cultures (solvent only). Data is represented as means of six technical replicates of *n* = 3 ± SD. *p < 0.05, One-Way ANOVA significantly below untreated solvent control.

Lithium chloride treatment of mouse ESC osteogenic cultures did not result in the establishment of an ID_50_ value (Fig. 2). Instead, calcification was dose-dependently up-regulated over control values in the absence of a cytotoxic effect. In contrast to mouse ESC cultures, LiCl induced a sharp drop in mitochondrial dehydrogenase activity, which is a routinely used and widely accepted test for cytotoxicity [3], [28], [29], in marmoset ESCs at a concentration of 100 μg/ml. LiCl-treated osteogenic marmoset ESC cultures also demonstrated a dose-dependent decrease in calcification as concentration increased with an ID_50_ at 0.4 ± 0.03 μg/ml, almost two orders of magnitude lower than the IC_50_. These results classify lithium chloride as teratogenic in marmoset, but not in mouse.

Comparatively, murine osteogenic cultures treated with sodium chloride featured consistently elevated calcification levels with a 3-fold increase observed in the lowest tested concentration (Fig. 2B). No reduction in calcification was observed in any tested concentration. Sodium chloride treated marmoset ESCs, in turn, displayed a half-maximal inhibitory dose at 790 ± 256 μg/ml. Sodium chloride-treated murine and marmoset osteogenic cultures followed relatively similar patterns of dose-dependent decreases in cell viability. In both cases, half maximal viability was approached, but not achieved within the test concentration range. The absent cytotoxicity coupled with a relatively high ID_50_ concentration qualified sodium chloride as non-cytotoxic and non-embryotoxic in neither species.

**Fig. 3:**
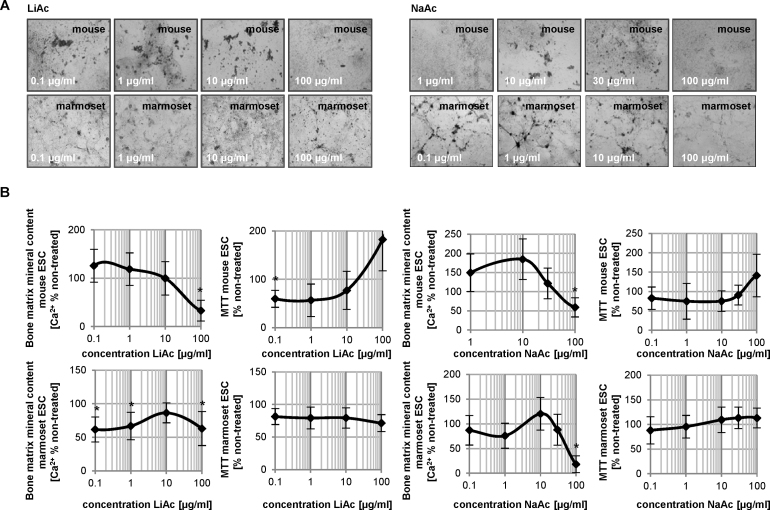
Cytotoxicity and bone mineral matrix assessment in mouse and marmoset osteogenic cultures treated with lithium and sodium acetate. (A) Photomicrographs of cultures treated with concentrations of compounds as indicated. (B) Cell viability and calcium deposit in treated cultures is graphed as a function of percent solvent control. Data is represented as means of three independent experiments, each including six technical replicates ± SD. *p < 0.05, one-way ANOVA significantly lower than untreated vehicle control.

Lithium acetate treatments resulted in a significant reduction in calcification at the highest tested concentration, while calcification levels remained above the 50% mark in marmoset ESCs for all concentrations tested (Fig. 3A and B). No decrease in cellular viability was noted upon exposure with this compound. In contrast, sodium acetate treatment induced a reduction in calcification at concentrations where viability was still around 100% (Fig. 3B). Although this effect was noted in both species, marmoset ESCs were more sensitive to sodium acetate than mouse ESCs.

### 3.3 Common sensitivity of mouse and marmoset ESCs to aluminum and controls

**Fig. 4:**
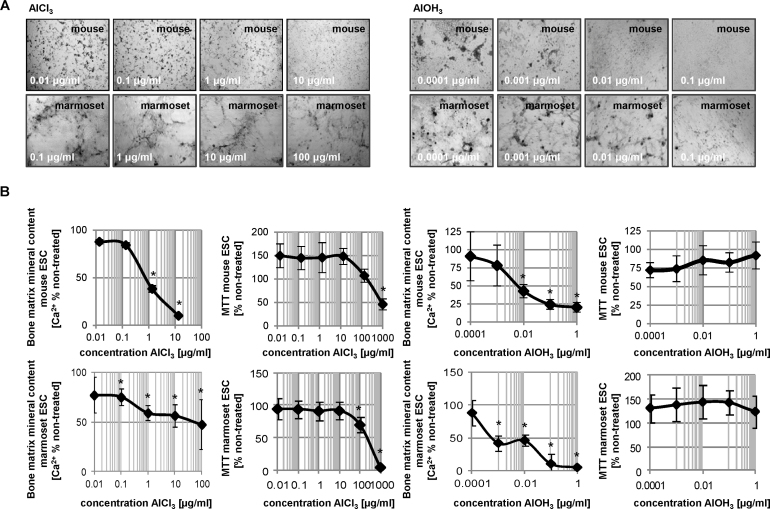
Cytotoxicity and differentiation inhibition in aluminum treated mouse and marmoset osteogenic ESC cultures. (A) Morphology on day 14 of differentiation. (B) Values measured for cell viability and calcium deposit were charted in percent of the untreated control. Data is represented as means of six technical replicates of *n* = 3 ± SD. *p < 0.05 below untreated vehicle control, one-way ANOVA.

In order to further assess response variation between mouse and marmoset ESC osteogenic cultures, effects on calcification levels were also investigated in aluminum chloride, another compound actively used in certain classes of pharmaceuticals with known detrimental effects on the developing skeleton [30], [31], [32]. Cells were treated with aluminum hydroxide as a control for the chloride in aluminum chloride. In aluminum chloride- and aluminum hydroxide-treated mouse ESC cultures, a dose-dependent decrease in calcification was observed with increasing concentration of the test compound (Fig. 4). A slightly steeper decrease in calcification was observed in aluminum hydroxide-treated cells. The similar response pattern between the aluminum chloride and hydroxide compounds infers that the observed teratogenic effect may be due to the presence of aluminum at those test concentrations. Comparable dose-dependent decreases in calcification were observed in marmoset ESC osteogenic cultures treated with aluminum chloride or hydroxide. However, calcification reduction in aluminum chloride-treated cultures was not as dramatic compared to osteogenic murine responses.

The similarity between mouse and marmoset ESC responses to treatment with aluminum compounds as summarized in Table 1, suggests the potential for response overlap between species utilized for *in vitro* teratogenicity assessments. However, whether or not this overlap occurs may depend on the chemical in question. Lithium is chemically similar to aluminum and yet did not produce similar responses between mouse and marmoset ESC cultures following treatment with lithium compounds (Table 1). Therefore, observed variations in response may be due to variability in species sensitivity to particular compounds.

**Table 1:** Half-maximal inhibitory concentrations of osteogenic differentiation (ID_50_) and cell viability (IC_50_) for chloride and aluminum compounds determined with mouse and marmoset ESCs.

Compound	ID_50_ (differentiation inhibition, Ca^2+^) [μg/ml]		IC_50_ (cytotoxicity, MTT) [μg/ml]	
	Mouse	Marmoset	Mouse	Marmoset
LiCl	n/a	0.4 ± 0.03	n/a	24.6 ± 14.8
NaCl	n/a	790 ± 256	n/a	n/a
LiAc	48 ± 11.3	n/a	n/a	n/a
NaAc	100 ± 21	52 ± 9.6	n/a	n/a
AlCl_3_	0.7 ± 0.02	80 ± 23.8	809 ± 137	204 ± 144
AlOH_3_	0.0055 ± 0.0035	0.0014 ± 0.0013	n/a	n/a

### 3.4 Differential sensitivity to skeletal teratogens in ESCs from Old and New World monkeys

Because of their close phylogenetic relationship with humans, primates share a large number of traits important in human reproduction. However, the reproductive biology of many small primates including *Callithrix*, is distinct from that of humans and Old World monkeys [33]. Because of the closer relationship between humans and Old World monkeys, we next investigated whether ESCs from the rhesus monkey, *Macaca mulatta*, showed similar responses to compounds as the marmoset ESCs. Rhesus ESCs are generally capable of responding to osteogenic triggers with enhanced matrix mineralization [17].

In order to compare the responsiveness of mouse, marmoset and rhesus ESCs to embryotoxic compounds, the embryotoxic potential of 5-fluorouracil (5-FU) and all-trans retinoic acid (*at*RA) in each species were compared against murine ESCs using the skeletal EST protocol [59]. Both compounds were previously shown to act as strong skeletal teratogens in the mouse, both *in vivo* and in the EST [3], [6], [14], [34], [35], [36], [37], [38]. Penicillin G (PenG) was included as a non-embryotoxic compound [21]. Effects on differentiation were assessed *via* calcium deposition quantification assay while cell viability was again measured with the MTT assay.

**Fig. 5:**
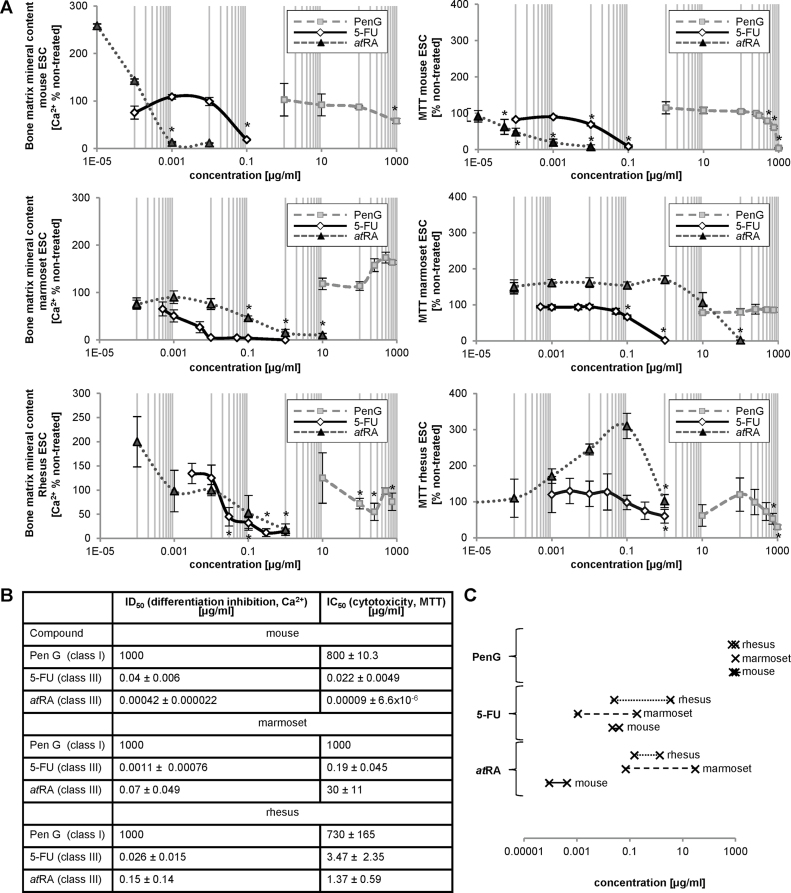
Comparison of marmoset and rhesus ESCs for their sensitivity to skeletal embryotoxicants. (A) Juxtaposition of cell viability and mineralization measurements taken from osteogenic mouse, marmoset and rhesus ESC cultures treated with *at*RA, 5-FU and PenG. *p < 0.05 below untreated vehicle control, one-way ANOVA. (B) Table contrasting the resulting IC_50_ and ID_50_ values taken from the concentration-response curves. (C) Chart depicting the concentration difference between IC_50_ and ID_50_ for each species grouped by compound. The left cross on each line indicates the respective ID_50_ value, the right cross the IC_50_ value.

In 5-FU-treated cells, similar decreases in cell viability were observed in mouse, marmoset and rhesus ESC osteogenic cultures (Fig. 5A). However, the mouse ESCs were the most sensitive to the cytotoxic effects of this compound, while marmoset and rhesus ESCs were equally sensitive. Measured calcification patterns in mouse, marmoset and rhesus ESC osteogenic cultures all followed dose-dependent decreases in mineralization with increased 5-FU concentration. Compared to the mouse, the marmoset cells were more sensitive, but the rhesus cells were less sensitive (Fig. 5B and C). However, in both primate cells, the ID_50_ was approximately two orders of magnitude lower that the IC_50_, indicating a strong teratogenic effect in both primate cell types.

Exposure to *at*RA treatment again caused cytotoxicity at lower concentrations in mouse ESC cultures than in both primate cell cultures. However, both mouse and rhesus ESC osteogenic cultures displayed a 2–2.5-fold increase in calcification at lower *at*RA concentrations, followed by a dose-dependent decrease in mineralization as concentration of *at*RA increased (Fig. 5A). Calcification levels in *at*RA-treated marmoset ESC osteogenic cultures resembled those of the untreated control at lower concentrations before gradually decreasing dose-dependently. Mouse ESC osteogenic cultures demonstrated the highest sensitivity as calcification at the highest tested concentration in the mouse ESC cultures was significantly lower than mineralization levels observed in marmoset and rhesus cultures at those same concentrations (Fig. 5B and C).

According to its role as a non-embryotoxic agent, PenG induced cytotoxicity only at high test concentrations, but failed to cause inhibition of calcification in all species (Fig. 5A–C). Results of this assay correctly identified 5-FU and *at*RA as strongly teratogenic compounds across the three species and PenG as non-teratogenic.

Observed differences in primate cell viability and calcification at higher concentrations of 5-FU and *at*RA compared to the mouse ESC osteogenic cultures suggest that both marmoset and rhesus ESCs may be less sensitive than mouse ESCs to particular classes of cytotoxic compounds, while they are more sensitive toward embryotoxic effects of others, again underlining our results obtained with the lithium and aluminum derivatives. Thus, murine-based cytotoxicity and *in vitro* skeletal embryotoxicity assays may provide limited predictivity for extrapolation of results to other species. Though these results indicate that both marmoset and rhesus ESC osteogenic cultures are capable of assessing cytotoxicity and embryotoxicity, the lack of a defined pattern of variability between primate osteogenic cultures indicates that response of non-human primate ESCs may also vary between different compound classifications.

## 4 Discussion

Since its introduction and subsequent validation, the classic EST has been updated to include additional tissue and molecular endpoints. Such revisions have proven to be extremely useful in allowing for reductions in assay duration as well as providing embryotoxicity responses across tissue types. Skeletal toxicity evaluations in particular stand much to gain from recent improvements as musculoskeletal birth defects account for 5% of all infant deaths. Previous work has demonstrated the capacity of the EST to identify inhibitory effects of toxicants on skeletal development based on the relationship between compound cytotoxicity as measured by reduction in mitochondrial dehydrogenase activity and inhibition of normal differentiation [6], [10].

In developmental toxicology, cytotoxicity of a chemical is often established with MTT assays and previous versions of stem cell based developmental toxicity assays also rely on this read-out measure [4], [39], [40]. However, strictly speaking the MTT assay is a measure for the mitochondrial activity of cells and is therefore only an indirect indicator of cytotoxicity. This could be of concern as mitochondrial activity in stem cells is different than in somatic cells. For instance, stem cells have a low number of mitochondria [41], which increases as cells differentiate concurrently with an increase in mitochondrial DNA content [42]. Future studies will need to compare different endpoints of cytotoxicity, such as apoptosis or proliferative capacity, for their predictivity *in vitro*, which is beginning to be done for other tissue endpoints [43].

One of the main drawbacks of the murine based EST is that it provides a narrow mechanistic understanding of human development and response to toxicants. Yet, potential human sources of cells are either ethically unaccepted in some countries or have been suggested to be of flawed quality. For example, human induced pluripotent stem cells (hiPSCs) often exhibit varying differentiation potential, due to altered global methylation or transcript number of master regulators, which greatly affects their quality and usability [44], [45], [46] and seems dependent on the choice of reprogramming factors [47]. As such, a proposed solution has been to update the EST to feature non-human primate ESCs as a basis for embryotoxicity assessment. Here, we applied a marmoset ESC-based EST in order to evaluate the efficacy of non-human primate ESCs in predicting potential negative side effects on the developing skeletal system. Our proof-of-concept results show that non-human primate ESCs and murine ESCs respond differently in embryotoxicity screens.

Our preliminary comparison screen of murine and non-human primate ESC-based EST assessments indicated that non-human primate ESCs were more tolerant toward the toxic effects of 5-FU and *at*RA compared to murine ESCs. Thus, it is possible that EST embryotoxicity results could be affected by species-driven response variation. Such variations may be attributed to differences in mouse and non-human primate molecular and genomic response to test compounds. Similar species-based discrepancies attributed to variations in molecular and genomic response have been observed in other studies [48]. Discrepancies between the effects of trauma, burns, and exposure to endotoxemia on temporal gene response patterns and inflammation signaling pathways were noted between mouse models and human patients. Though responses were similar among human subjects, comparison of mouse and human results showed poor correlation of responses between the two groups at the molecular and genomic levels. As all compounds function at the molecular and/or genomic level, these results call into question the extrapolation efficacy of mouse responses as predictors of response in humans [49], [50]. Given the evolutionary closeness between humans and non-human primates [51], it is probable that a mechanism of variation similar to that seen in the Seok et al. [48] study is operating in this study between the murine ESC and marmoset ESC cultures.

Aluminum chloride assay results in murine and marmoset ESCs both demonstrated dose-dependent decreases in calcification. As decreases were observed in both AlCl_3_- and AlOH_3_-treated cultures, it is probable that Al^3+^ is responsible for the osteotoxic effects of AlCl_3_ exposure. Recent studies on aluminum osteotoxicity in infants have reported a strong connection between pre- and perinatal aluminum overexposure and metabolic bone diseases as well as potential long term consequences on bone health and development following exposure to aluminum compounds during critical periods of development [30]. At the molecular level, aluminum has been suggested to antagonize bone formation through activation of the oxidative-stress-mediated c-Jun N-terminal kinase signaling pathway and subsequent induction of apoptosis in osteoblasts [52].

Overall evaluation of cell viability and calcification assay results indicated that lithium chloride and its derivatives possess skeletal teratogenic capacity, though the potency of teratogenic effects may vary depending on the other members of the lithium compound complex. Treatment of marmoset ESC osteogenic cultures with both lithium chloride and lithium acetate compounds demonstrated noteworthy, but species-specific decreases in calcification, which were absent in sodium chloride. In contrast, sodium acetate was teratogenic in both species. These results suggest lithium to not be the chief skeletal teratogenic component, but rather the combination with the complexed chemical that results in the detrimental outcome on differentiating osteoblasts. Additionally, varied response to lithium and sodium compound treatment between murine and marmoset ESC cultures suggests that species variation in embryotoxic assessments may yield a varied and potentially narrow scope of responses to the compounds under investigation.

Within the cell, lithium chloride has been suggested to operate *via* inhibition of glycogen-synthase-kinase 3beta (GSK3β) to intensify canonical Wnt signaling, which ultimately encourages upregulation of genes required for osteogenic differentiation. Previous studies have reported dose-dependent bone defects incurred by disruption of the canonical Wnt signaling pathway with lithium chloride and support the dose dependent effects of lithium chloride treatment on mouse ESC cultures reported here [53], [54].

Conversely, marmoset ESCs were much more sensitive to the detrimental effects of lithium chloride. Although this may be largely due to increased cytotoxicity of lithium chloride at higher concentrations, the differentiation effect occurred at concentrations that were two orders of magnitude lower than the cytotoxic effect. Considering that other studies have reported variations between mouse and human response at the molecular level, it is likely that a similar explanation stands for the observed differences in lithium chloride response in this study. Lithium chloride is often included in embryotoxicity screens as a control compound in the class of the moderate embryotoxicants [21]. Intriguingly, the classical EST has a low prediction value for the moderate embryotoxicants [55] and it stands to reason that this low predictivity in this specific class of teratogens stems at least partially from using a less predictive species such as the mouse instead of primate cells.

Of concern are our findings on the differential sensitivity between marmoset and rhesus ESCs that exist even in the small set of chemicals tested here. Although the three control chemicals that were tested exhibited predictive effects, with 5-FU and *at*RA being teratogenic and PenG not, the actual half-maximal inhibitory doses varied substantially. It is thus evident that there may be significant general differences in the responses of different non-human primate cells to drugs and toxicants in a broader screen encompassing more chemicals as is typically done with mouse cells [6], [11]. However, our limited results already suggest that this existing dose discrepancy may make risk predictions for human use and the definition of adverse outcome doses difficult. For the reasons laid out in this manuscript, human embryonic stem cells, which are already beginning to be explored for such purpose [56], [57], [58], may provide the most accurate information regarding the teratogenic potential of chemicals and future studies will need to show whether the ethically accepted human induced pluripotent stem cells are also predictive in such assays.

## Conflict of interest

The authors have no conflict of interest to declare.

## Transparency document

Transparency document
